# Nonmuscle Myosin IIA Regulates the Precise Alignment of Hexagonal Eye Lens Epithelial Cells During Fiber Cell Formation and Differentiation

**DOI:** 10.1167/iovs.64.4.20

**Published:** 2023-04-18

**Authors:** Sadia T. Islam, Catherine Cheng, Justin Parreno, Velia M. Fowler

**Affiliations:** 1 Department of Biological Sciences, University of Delaware, Newark, Delaware, United States; 2 School of Optometry and Vision Science Program, Indiana University, Bloomington, Indiana, United States; 3 Department of Molecular Medicine, The Scripps Research Institute, La Jolla, California, United States

**Keywords:** Myh9, lens meridional rows, epithelial cell patterning, hexagonal cell shape

## Abstract

**Purpose:**

Epithelial cells in the equatorial region of the ocular lens undergo a remarkable transition from randomly packed cells into precisely aligned and hexagon-shaped cells organized into meridional rows. We investigated the function of nonmuscle myosin IIA (encoded by *Myh9*) in regulating equatorial epithelial cell alignment to form meridional rows during secondary fiber cell morphogenesis.

**Methods:**

We used genetic knock-in mice to study a common human *Myh9* mutation, E1841K, in the rod domain. The E1841K mutation disrupts bipolar filament assembly. Lens shape, clarity, and stiffness were evaluated, and Western blots were used to determine the level of normal and mutant myosins. Cryosections and lens whole mounts were stained and imaged by confocal microscopy to investigate cell shape and organization.

**Results:**

We observed no obvious changes in lens size, shape, and biomechanical properties (stiffness and resilience) between the control and nonmuscle myosin IIA–E1841K mutant mice at 2 months of age. Surprisingly, we found misalignment and disorder of fiber cells in heterozygous and homozygous mutant lenses. Further analysis revealed misshapen equatorial epithelial cells that cause disorientation of the meridional rows before fiber cell differentiation in homozygous mutant lenses.

**Conclusions:**

Our data indicate that nonmuscle myosin IIA bipolar filament assembly is required for the precise alignment of the meridional rows at the lens equator and that the organization of lens fiber cells depends on the proper patterning of meridional row epithelial cells. These data also suggest that lens fiber cell organization and a hexagonal shape are not required for normal lens size, shape transparency, or biomechanical properties.

The ocular lens is a transparent cellular organ whose main function is to fine focus light onto the retina to transmit a clear image.^1^^,^^2^ The lens is surrounded by a basement membrane, called the capsule, and is composed of two types of cells, a monolayer of epithelial cells at the anterior surface overlying a bulk mass composed of fiber cells (Fig. 1A). Near the lens equator, epithelial cells in the germinative zone proliferate, and equatorial epithelial cells undergo a remarkable transformation from randomly packed, cobblestone-shaped cells to precisely aligned and hexagon-shaped cells, forming the meridional row cells (Fig. 1B) that further differentiate and elongate into secondary fiber cells.^3^^–^^8^ Secondary fiber cells elongate more than 1000-fold, yet retain morphological characteristics from the meridional row cells, including precise alignment, tight packing, and hexagon cell shape (in equatorial cross-section).^2^^,^^8^

**Figure 1.**
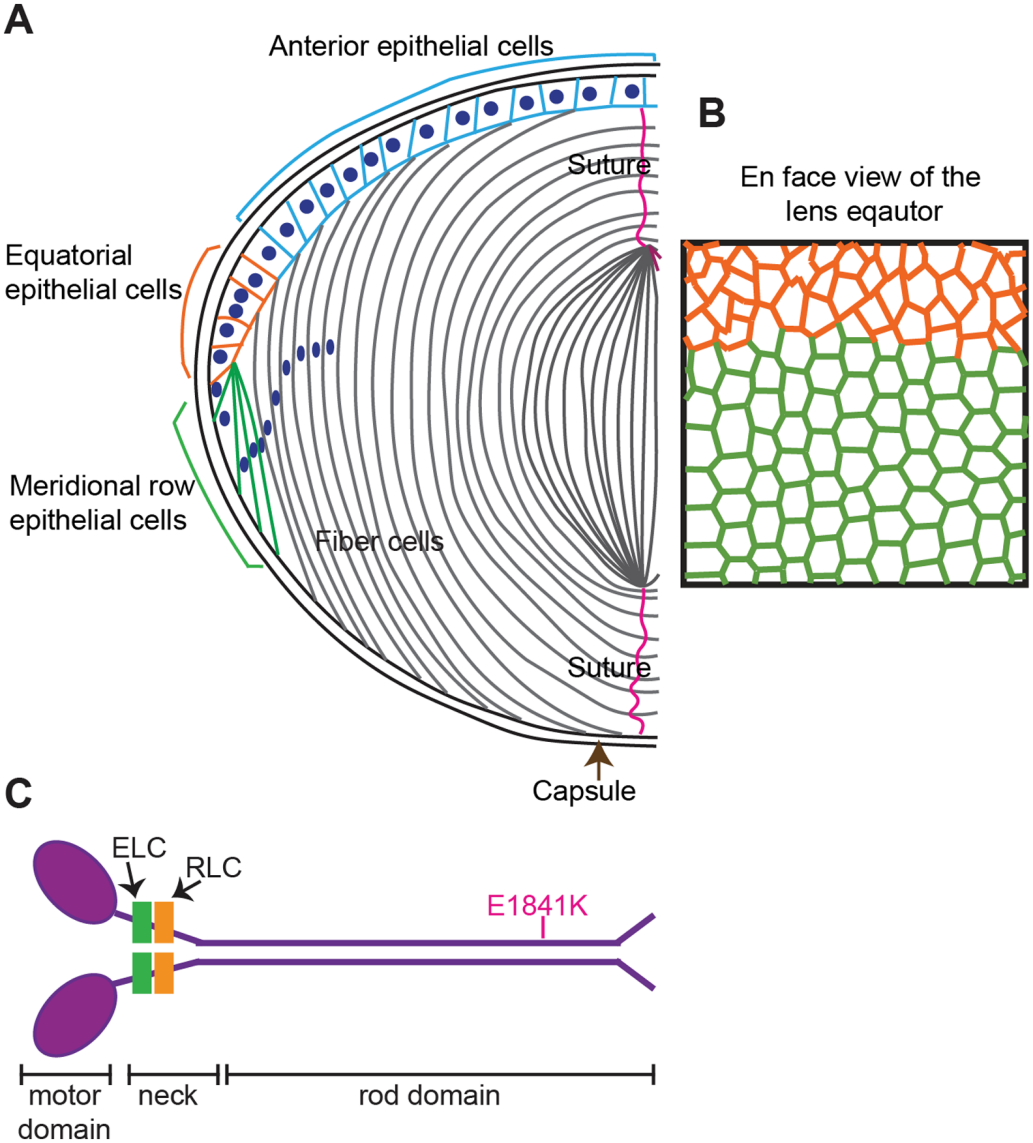
Diagrams of mouse lens anatomy and NMIIA structure. (**A**) A cartoon of a sagittal view of the mouse lens. The ocular lens is composed of two types of cells, epithelial cells (*colored*) and fiber cells (*gray*). The anterior epithelial cells are quiescent (*blue*), whereas the equatorial epithelial cells (*orange*) have proliferative activity and can migrate further down to the equator to differentiate into meridional row epithelial cells (*green*). The meridional row epithelial cells further differentiate into secondary fiber cells (*grey*). (**B**) A cartoon of the en face view of the lens equator shows that the irregularly shaped and randomly packed equatorial epithelial cells (*orange*) become precisely aligned, hexagon shaped and arranged in a honeycomb pattern (*green*). The green cells are arranged into meridional rows, which further elongate and differentiate into secondary fiber cells (*gray*). (**C**) NMIIA molecules are hexamers containing two heavy chains (*purple*), each consisting of an N-terminal motor domain with actin-activated ATPase activity, a flexible neck, and a rod domain. The essential light chain (ELC) (*green*) and regulatory light chain (RLC) (*orange*) bind to the heavy chain in the neck region. NMIIA activity is increased or decreased by RLC phosphorylation or dephosphorylation, respectively. The E1841K mutation (*pink*) in the rod domain is one of the most common *MYH9-*RD mutations in humans. Cartoons not drawn to scale.

The mechanisms that regulate lens meridional row alignment are not well-understood, with the only known pathway being signaling through the receptor tyrosine kinase EphA2.^8^^,^^9^ Eph receptors are the largest class of receptor tyrosine kinases that regulate cell contact–dependent communication, adhesion, and migration.^10^^,^^11^ Previous studies have shown that the EphA2 signaling pathway regulates hexagon cell shape and alignment of meridional row cells, as well as the subsequent ordered packing of fiber cells via signaling to the actin cytoskeleton.^8^^,^^9^^,^^12^^,^^13^

In other tissues, actomyosin contractility regulates the hexagonal packing of epithelial sheets and modulates cell shape changes during development.^14^^–^^17^ Nonmuscle myosin IIs (NMIIs) are conserved actin-binding proteins ubiquitously expressed in various mammalian tissues in a tissue-specific combination of three paralogs, NMIIA, NMIIB, and NMIIC.^18^^,^^19^ NMII forms bipolar filaments that crosslink and slide antiparallel actin filaments (F-actin), contracting F-actin into actomyosin bundles.^18^^,^^20^^–^^22^ NMII–F-actin contractility regulates cellular processes, such as cell shape, polarity, migration, and cytokinesis, all of which are critical processes for tissue morphogenesis.^21^^,^^23^^,^^24^ The functions of myosin II in epithelial cell hexagonal packing, alignment, and cellular rearrangements have been studied previously in *Drosophila* embryonic epithelial sheets and mammalian cochlear ducts.^17^^,^^25^^–^^33^ The mouse ocular lens is an excellent model system to study morphogenesis and epithelial cell alignment because of the accessible location of the lens epithelium on the surface, and the lifelong addition of precisely aligned hexagonal epithelial cells during the continuous differentiation of the meridional row epithelial cells to fiber cells.^2^^,^^6^^,^^34^^–^^38^

NMIIA and NMIIB are encoded by the *Myh9* and *Myh10* genes, respectively, and are abundant in the mouse lens epithelium, whereas NMIIC (encoded by the *Myh14* gene) has significantly lower expression in the lens compared with the other NMII isoforms.^39^ Low expression levels of NMIIA and NMIIB are also observed in lens fiber cells, where weak immunostaining is observed colocalizing with F-actin at the short vertices of the fiber cells in cryosections of mature mouse lens.^39^^,^^40^ Mutations in the human *MYH9* gene can cause cataracts and are also associated with various tissue pathologies, including thrombocytopenia, platelet macrocytosis, proteinuric neuropathy, and sensorineural deafness.^41^^–^^46^ These disorders are collectively called *MYH9*-related diseases (*MYH9-*RD).^41^^,^^42^^,^^47^^–^^49^ More than 40 different mutations in *MYH9* are associated with *MYH9-*RD in human patients.^24^ One of the most common *MYH9* mutations is E1841K in the rod domain (Fig. 1C).^41^^,^^42^^,^^50^^–^^52^ Mouse models with NMIIA knock-in mutations for E1841K recapitulate the human disease with low platelet counts, neutrophil inclusions, kidney abnormalities, hearing loss, and lens opacitites.^50^^,^^53^^,^^54^

Previous work from our laboratory and others shows that the NMIIA–E1841K mutation causes abnormal bipolar filament formation.^55^^,^^56^ Rotary shadowing electron microscopy of purified NMIIA filaments indicates that mutant NMIIA–E1841K bipolar filaments are abnormal^55^ with longer and thicker bipolar filaments.^55^ NMIIA–E1841K mutant bipolar filaments also displayed myosin heads (motors) all along their length, leading to the loss of the central bare zone and disruptions in filament bipolarity.^55^ The bare zone is normally in the middle of bipolar filaments where the rod domains associate with each other, and myosin heads are not present in that region.^57^ This aberrant NMIIA–E1841K filament organization most likely contributes to the misaligned actomyosin structures that are observed in megakaryocytes from mice with the NMIIA–E1841K mutation.^55^

Here, we used the NMIIA–E1841K knock-in mouse to study the lens phenotype and investigate whether disruption of NMIIA filament assembly by the E1841K mutation leads to changes in lens transparency, size, shape, and/or biomechanical properties. We also determined the effect of the NMIIA–E1841K mutation on lens cellular organization, in particular the alignment and hexagonal packing of secondary fiber cells and meridional row epithelial cells near the lens equator. Our data demonstrate that the NMIIA-E1841K mutation has no effect on whole lens size, shape, or biomechanical properties, but leads to impaired equatorial epithelial cell alignment into meridional row cells during fiber cell morphogenesis. Subsequently, there is disordered fiber cell packing in mutant lenses. Thus, this work suggests that normal NMIIA bipolar filaments are required for the organization of lens equatorial epithelial cells into neatly packed meridional rows, and are consistent with previous studies showing that the organization of the meridional rows is required for normal fiber cell alignment and shape.^8^^,^^9^

## Methods

### Mice

All animal procedures were conducted in adherence to the ARVO Statement for the Use of Animals in Ophthalmic and Vision Research and performed in accordance with approved animal protocols from the Institutional Animal Care and Use Committee guidelines at the Scripps Research Institute and the University of Delaware. Genetic knock-in mice with the *Myh9*–E1841K mutation^53^ were obtained from Dr. Robert Adelstein (National Heart, Lung, and Blood Institute, National Institutes of Health, Bethesda, MD).

We intercrossed NMIIA*^E1841K/+^* mice to generate NMIIA*^+/+^*, NMIIA*^E1841K/+^*, and NMIIA*^E1841K/E1841K^* littermate mice. The E1841K strain was created in a mixed strain background of FvBN/129SvEv/C57Bl6. The FvBN strain carries an endogenous mutation in the *Bfsp2* gene that causes a spontaneous knockout of CP49, a beaded intermediate filament protein that is critical for maintaining mature fiber cell morphology and whole lens biomechanical properties.^58^^–^^61^ Therefore, we backcrossed the NMIIA*^E1841K/E1841K^* mice with C57BL6/J wild-type mice and screened the offspring for the presence of the wild-type CP49 allele.^61^ Mice were maintained with two copies of wild-type CP49. Intercrosses of NMIIA*^E1841K/+^* mice over 2 years produced mice with 35% NMIIA*^+^^/+^*, 53% NMIIA*^E1841K/+^*, and 12% NMIIA*^E1841K/E1841K^* genotypes (Supplementary Table S1). The number of homozygous NMIIA*^E1841K/E1841K^* pups per litter (12%) was lower than expected from Mendelian genetics (approximately 25%), suggesting that mice homozygous for the NMIIA–E1841K mutation have a survival disadvantage, consistent with previous results.^50^

### Lens Morphometrics and Biomechanical Testing

Lenses from 2-month-old littermate control and mutant mice were dissected immediately from freshly enucleated eyeballs at room temperature in 1× phosphate buffered saline (PBS, 14190, Thermo Fisher Scientific, Grand Island, NY, USA). Lens pictures were acquired with an Olympus SZ11 dissecting microscope using a digital camera (Nikon Coolpix 995). Lens axial and equatorial diameters were measured on acquired images using FIJI software. To calculate lens volume, we used the formula, volume = 4/3 × π × r_E_2 × r_A_, where r_E_ is the equatorial radius and r_A_ is the axial radius.^37^^,^^40^^,^^62^^,^^63^ The lens aspect ratio was calculated by dividing the equatorial diameter by the axial diameter.^61^^,^^63^ Eight to 10 lenses from 4 to 5 mice were used for morphometric analysis. Lenses from 2-month-old mice were imaged on a 200-mesh grid (Electron Microscopy Sciences, Hatfield, PA, USA; Catalog G300H-Cu) in 1× PBS under light- and dark-field optics (Zeiss Stemi SV dissecting microscope; Carl Zeiss Meditec, Jena, Germany). The materials for grid imaging were provided by Dr. Salil Lachke (University of Delaware).

Lens biomechanics were tested by the sequential application of glass coverslips, as previously described.^37^^,^^61^^–^^63^ Briefly, freshly dissected lenses were transferred to a custom chamber filled with 1× PBS and sequentially compressed with glass coverslips (≤10 coverslips; Fisherbrand Coverslip #1 catalog 12-542-AP, Thermo Fisher Scientific, Waltham, MA, USA; 18 mm × 18 mm; 129.3 mg each), followed by removal of the coverslips to examine lens recovery after load removal. Side view images of uncompressed and compressed lenses were obtained via a 45° angle mirror. Measurements of the lens axial and equatorial diameters were performed using FIJI. To calculate either axial or equatorial strain, we used the formula ε = (d − d_0_)/d_0_, where ε is strain, d is the axial or equatorial diameter at a given load, and d_0_ is the corresponding axial or equatorial diameter at zero load. Recovery of lens shape after compression (resilience) was measured as the ratio between the precompression and postcompression axial diameters. Six lenses from three mice of each genotype were used for biomechanical analysis.

### Gel Electrophoresis and Western Blots

Western blots were performed on lenses isolated from 6- to 8-week-old mice, as previously described.^40^ Lenses were dissected from freshly enucleated eyes and stored at –80°C until homogenization. Two lenses from each mouse were pooled into one protein sample. At least three pairs of lenses of each genotype were used to make separate protein samples. Lenses were homogenized on ice in a glass Dounce homogenizer in 250 µL of lens homogenization buffer (20 mM Tris-HCl pH 7.4 at 4°C, 100 mM NaCl, 1 mM MgCl_2_, 2 mM EGTA and 10 mM NaF with 1 mM DTT, 1:100 Protease Inhibitor Cocktail [P8430, Sigma-Aldrich, St. Louis, MO, USA], and 1:1000 Phosphatase Inhibitor [78420, Thermo Fisher Scientific] added on the day of the experiment) per 10 mg of lens wet weight. The lysates in the homogenization buffer were then diluted in 1:1 with a 2× Laemmli sample buffer (1610737, Bio-Rad Laboratories, Hercules, CA, USA). Samples were briefly sonicated with a Q55 Sonicator (Qsonica, Newtown, CT, USA) and boiled for 5 minutes. Proteins were separated on a 4% to 20% linear gradient SDS-PAGE mini-gels (XP04205BOX, Thermo Fisher Scientific) and transferred to nitrocellulose membranes (10600011, Amersham Protran, Slough, UK) at 150 V in 1× transfer buffer (25 mM Tris, 192 mM glycine in ddH_2_O) with 20% methanol + 0.1% SDS (myosin buffer^40^^,^^55^) in a trans-blot tank (Bio-Rad) at 4°C for 1 hour. Membranes were then stained with Ponceau S (09189, Fluka BioChemica, Mexico City, Mexico), and gently washed with ddH_2_O until the protein bands were pink and the surrounding membrane was white. The blots were scanned with a Bio-Rad Chemidoc MP to reveal total protein levels in each lane. Blots were blocked with 5% BSA in 1× PBS for 1 hour at room temperature. The blots were then incubated with primary antibodies diluted in 5% BSA + 0.1% Triton X-100 in 1× PBS overnight at 4°C with gentle rocking. For primary antibodies, we used anti-NMIIA (ab55456, 1:1000, Abcam, Cambridge, UK) and anti–nonmuscle myosin IIB (anti-NMIIB, M7939, 1:1000, Sigma-Aldrich). The blots were then washed with PBST (1× PBS + 0.1% Triton X-100, 3 × 5 minutes/wash) before incubation in secondary antibodies diluted with 5% BSA + 0.1% Triton X-100 in 1× PBS for 2 hours at room temperature in the dark with gentle rocking. Secondary antibodies (1:20,000 dilution) were IRDye-680LT-conjugated goat anti-mouse IgG (926-68020, LI-COR, Lincoln, NE, USA) and IRDye-800CW-conjugated goat anti-rabbit IgG (926-32211, LI-COR). After secondary antibody incubation, blots were washed again with PBST (4 × 5 minutes/wash). The band intensities of the blot were quantified using ImageJ with background subtraction and then normalized to the total protein level (Ponceau S staining).

### Immunostaining of Frozen Cryosections

Frozen lens sections from 6-week-old mice were prepared as previously described.^40^^,^^64^ Briefly, a small opening was made at the corneal–scleral junction of freshly dissected eyeballs to allow penetration of fixative. Eyeballs were fixed in freshly made 1% paraformaldehyde (15710, Electron Microscopy Sciences, Hatfield, PA, USA) in 1× PBS at 4°C for 4 hours. Samples were washed briefly twice in ice-cold 1× PBS, cryoprotected in 30% sucrose in 1× PBS for 3 to 4 hours until the eyes sank to the bottom of the tube, and embedded in OCT medium (Sakura Finetek, Torrance, CA, USA) in the cross-sectional orientation. Frozen blocks were stored at −80°C until sectioning. Equatorial cryosections (approximately 12 µm thick) were obtained with a Leica CM1950 cryostat and collected on glass slides. The sections were stored at –20°C until further use. Sections were rehydrated twice in PBST for 1 minute, permeabilized in 1× PBS with 0.3% Triton X-100 for 30 minutes, and blocked with 3% BSA, 1% goat serum, and 0.1% Triton X-100 in 1× PBS (blocking buffer) for 1 hour. Lens sections were labeled with mouse anti-NMIIA heavy chain (raised against NMIIA rod/tail domain) primary antibody (ab55456, 1:200, Abcam) in blocking buffer overnight at 4°C, washed three times for 5 minutes per wash in PBST, and then labeled for 1.5 to 2.0 hours with fluorescent-conjugated secondary antibody, rhodamine-phalloidin for F-actin (R415, 220 nM, Thermo Fisher Scientific), and Hoechst 33342 (62249, 1:1000 dilution, Thermo Fisher Scientific) for nuclei. Alexa-Fluor-647-conjugated goat anti-mouse IgG (A21236, Thermo Fisher Scientific) was used as the secondary antibody (1:200 dilution). The sections were then washed three times for 5 minutes per wash in PBST. ProLong Gold antifade reagent (Thermo Fisher Scientific) was used to mount coverslips on the slides. Imaging was performed using a Zeiss 780 laser-scanning confocal microscope (20× objective, NA 0.75 or a 100× objective, NA 1.4), or a Zeiss 880 laser-scanning confocal microscope (20× objective, NA 0.8 or 63× oil objective, NA 1.4). The equatorial region in the lens cross-sections was identified based on the thickness of the lens epithelium.^65^

### Whole-Mount Staining and Imaging of Fixed Lenses

Freshly dissected whole lenses were fixed by immersing in 4% paraformaldehyde in 1× PBS at room temperature for 1 hour. Fixed lenses were washed in 1× PBS (3 × 5 minutes) and labeled overnight at 4°C with 220 nM rhodamine-phalloidin (Thermo Fisher Scientific), CF®640R WGA (Biotium, 1:250), and Hoechst 33342 (1:500) in permeabilization/blocking solution (3% BSA, 3% goat serum and 0.3% Triton). After overnight incubation, lenses were washed in 1× PBS (3× for 5 minutes/wash) before imaging the lens epithelium and fiber cells by confocal microscopy. To prevent movement of lenses during imaging, lenses were immobilized in FluoroDish cell culture dishes (FD35-100, WPI) within a triangular divot that was created using a disposable razor blade, in a thin layer of 4% agarose in PBS.^8^^,^^37^^,^^62^ The whole mount z-stacks were acquired at a digital zoom of 1.0 with z-step sizes of 0.5 µm (20× objective) and 0.25 µm (63× objective). All images were processed in the Zen software (Carl Zeiss) for further analysis.

### Image Analysis

To measure fiber cell disorder, single optical section images of F-actin–labeled equatorial cryosections that were acquired with a 20× objective were used to investigate fiber cell organization. Disordered fiber cell patches were outlined manually, and disordered areas were measured using FIJI.^61^ Three different sections from three 3 different mice per genotype were used for fiber cell disorder analysis (for a total of nine sections). We measured peripheral fiber cell morphology in whole mount images at a standardized depth by identifying the fulcrum region of the lens, where the apical tips of elongating epithelial cells constrict to form an anchor point before fiber cell differentiation and elongation at the equator.^9^^,^^62^^,^^66^^,^^67^ Peripheral fiber cell morphology, revealed by F-actin and WGA staining, was determined at the lens equator, approximately 5.0 to 5.5 µm inward from the fulcrum.^62^ Raw images were processed and exported to FIJI. Four lens images from four biological replicates for NMIIA*^+/+^* lenses and eight lens images from five biological replicates for NMIIA*^E1841K/E1841K^* lenses were observed to determine if peripheral fiber cells seemed to be irregular in the NMIIA*^E1841K/E1841K^* lenses compared with control lenses.

The percent disordered area in the region of the meridional rows was measured from single optical sections from a z-stack of a lens whole mount acquired with a 20× objective. A single optical section of the meridional row cells approximately 5 µm peripheral to the fulcrum (toward the lens capsule) was selected for each lens. The entire region of the meridional row cells was outlined (region of interest), and the disordered areas were manually outlined in FIJI. The area of the disordered patches was divided by the total region of interest area to calculate the percent of disordered area. Four to eight different lens images from at least four different mice per genotype were used for this analysis (four lens images from four NMIIA*^+/+^* mice and eight lens images from five NMIIA*^E1841K/E1841K^* mice). The average disordered area and standard deviation were calculated and plotted in GraphPad Prism 9.

To determine the hexagonal packing of the meridional row cells, we identified an optical section at the basal region of the meridional row cells immediately below the lens capsule where all cells are in focus and on the same plane. We manually traced the boundary of each meridional row cell at the basal region and counted the number of adjacent cells. Forty to fifty cells from five different lens images were analyzed for each genotype (five lens images from four NMIIA*^+/+^* mice and five lens images from five NMIIA*^E1841K/E1841K^* mice). The frequency distribution and the average percentage of hexagonal and six adjacent cells were calculated and plotted in GraphPad Prism 9.

### Statistical Analyses

The mean, SD, and frequency distribution were all calculated and plotted. One-way ANOVA and two-tailed Student *t*-tests were performed in GraphPad Prism 9 for statistical significance.

## Results

### Lenses From Mice With the NMIIA–E1841K Mutation Are Transparent With Normal Focusing Ability, Shape, Size, and Biomechanical Properties

**Figure 2.**
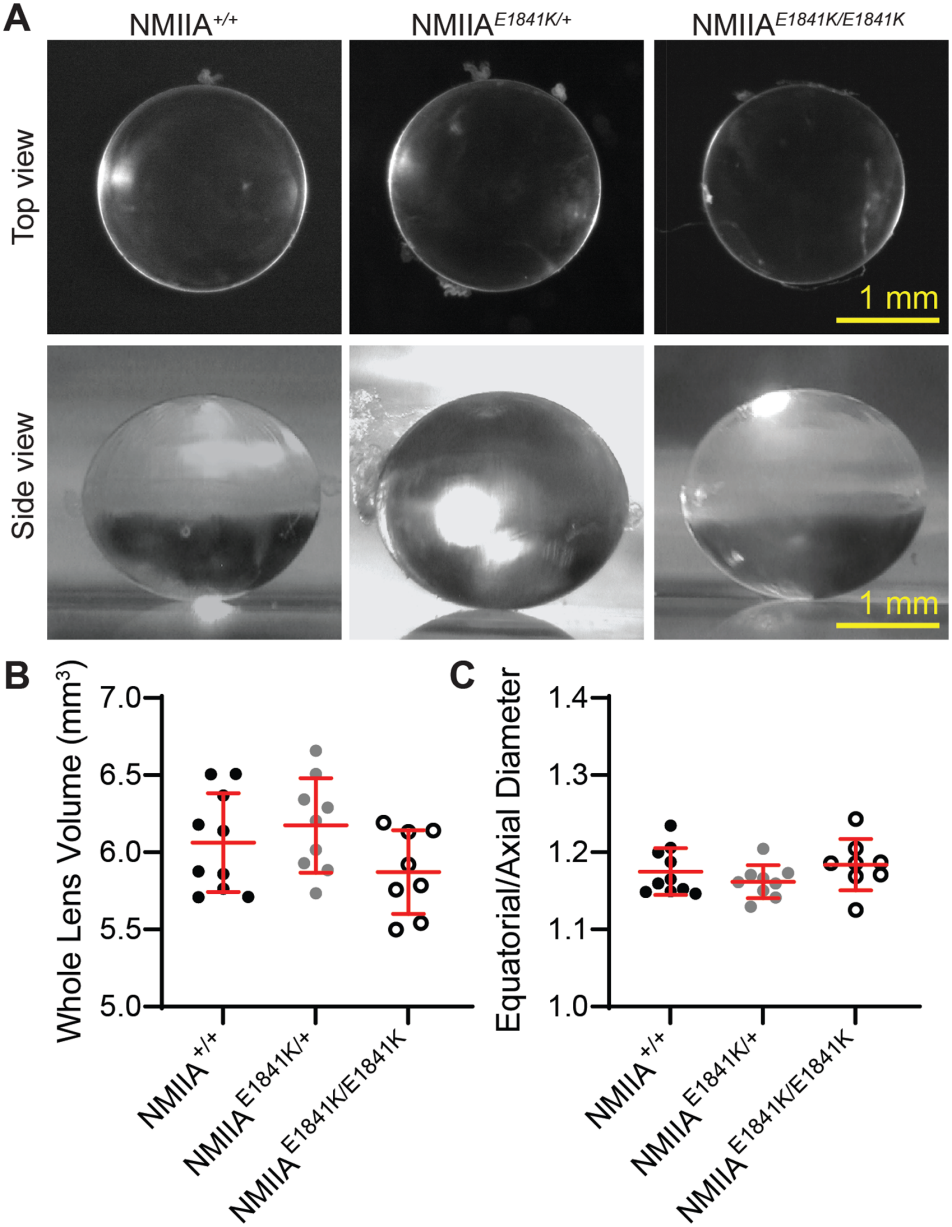
The NMIIA E1841K mutation has no effect on whole lens size and shape. (**A**) (*Upper panel*) Top-view images of freshly dissected 2-month-old NMIIA*^+/+^*, NMIIA*^E1841K/+^*, and NMIIA*^E1841K/E1841K^* lenses. All lenses were transparent without obvious opacities. Lower panel, side view images of lenses. Scale bars, 1 mm. (**B**) Whole lens volume and (**C**) aspect ratio (equatorial to axial diameter ratio) from 2-month-old control and mutant mice show no significant differences in whole lens size and shape. Plots reflect mean ± SD of 8–10 lenses from 4–5 biological replicates per genotype.

Lenses from 2-month-old control and NMIIA–E1841K mutant mice were transparent with no loss of clarity or apparent cataracts (Fig. 2A). To test focusing ability, we placed control and mutant lenses on electron microcopy grids. Both the control and NMIIA–E1841K mutant lenses are able to focus on the electron microscopy grid below the lens, and no qualitative differences in focusing ability were observed between control and mutant lenses (Supplementary Fig. S1A). Whole lens volume and aspect ratio (equatorial to axial diameter ratio) from 2-month-old mice were also calculated. The mean lens volumes of 2-month-old NMIIA*^+/+^*, NMIIA*^E1841K/+^*, and NMIIA*^E1841^^K^^/E1841K^* mice were 6.06 ± 0.32 mm^3^, 6.17 ± 0.31 mm^3^, and 5.87 ± 0.27 mm^3^, respectively, and the mean lens aspect ratios of 2-month-old NMIIA*^+/+^*, NMIIA*^E1841K/+^* and NMIIA*^E1841^^K^^/E1841K^* mice were 1.17 ± 0.03, 1.16 ± 0.02, and 1.18 ± 0.33, respectively (Figs. 2B and 2C). The morphometric analysis of 2-month-old lenses shows no significant changes in volume and aspect ratio between control, heterozygous, and homozygous mice (Figs. 2B and 2C).

Previous studies have suggested that myosin II activity influences whole lens biomechanics.^68^^,^^69^ Therefore, we measured lens stiffness using a coverslip compression method in which coverslips are sequentially loaded onto individual lenses resulting in axial compression and equatorial expansion of the lens from which the strain can be calculated (Supplementary Fig. S1B).^61^^–^^63^ Lens resiliency (recovery after load removal) was also measured. No significant differences in lens stiffness or resiliency were observed between control, heterozygous, and homozygous lenses from 2-month-old mice (Supplementary Fig. S1B).

### NMIIA–E1841K Mutant Proteins Form Intracellular Puncta in Lens Fibers

**Figure 3.**
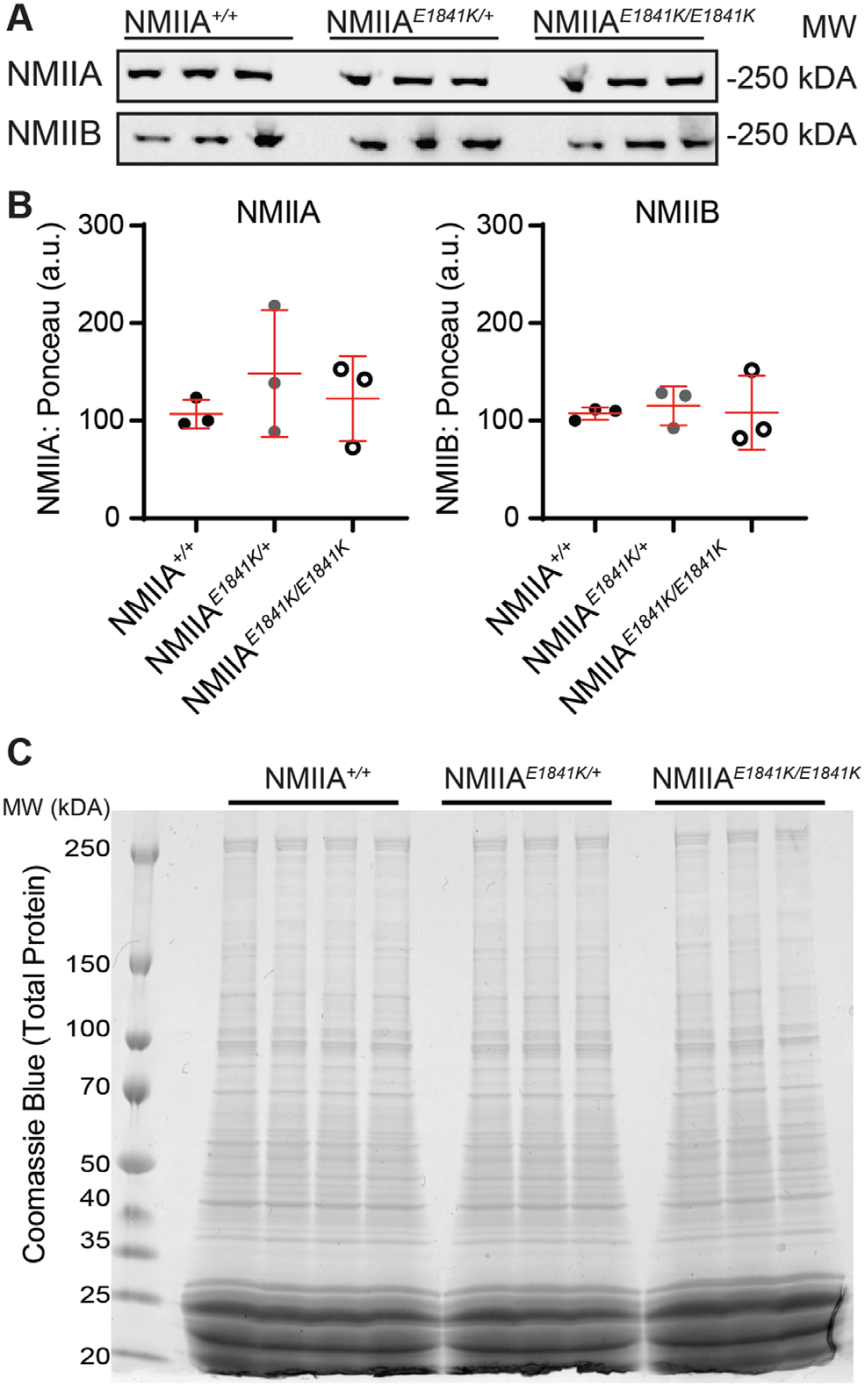
The NMIIA-E1841K mutation does not affect the global expression of NMIIA/B isoforms in the lens. (**A**) Western blots of NMIIA and NMIIB heavy chains in whole lens lysates from 6- to 8-week-old NMIIA*^+/+^*, NMIIA*^E1841K/+^*, and NMIIA*^E1841K/E1841K^* mice. (**B**) Relative NMIIA/B heavy chain expression in NMIIA*^+/+^*, NMIIA*^E1841K/+^*, and NMIIA*^E1841K/E1841K^* whole lenses. NMIIA and NMIIB heavy chain protein levels were normalized to total protein level (Ponceau S staining), which shows no significant changes in protein expression level in the NMIIA-E1841K mutant lenses compared with control lenses. Plots reflect the mean ± SD of *n* = 3 biological replicates per genotype. *P < 0.05. (**C**) Coomassie blue staining of total lens extracts from 6- to 8-week-old NMIIA*^+/+^*, NMIIA*^E1841K/+^*, and NMIIA*^E1841K/E1841K^* mice. No noticeable changes in total protein levels were observed between control and mutant lens lysates. Three or four biological replicates were tested for each genotype.

The mouse lens expresses two NMII isoforms, NMIIA and NMIIB.^39^ Western blots of whole lens extracts indicate that NMIIA and NMIIB protein levels in NMIIA*^E1841K/+^* and NMIIA*^E1841^^K^^/E1841K^* heterozygous and homozygous mutant lenses are comparable with control NMIIA*^+/+^* lenses (Figs. 3A and B). Coomassie blue staining of protein gels shows no obvious changes in total protein levels in mutant E1841K lenses compared with control lenses (Fig. 3C).

**Figure 4.**
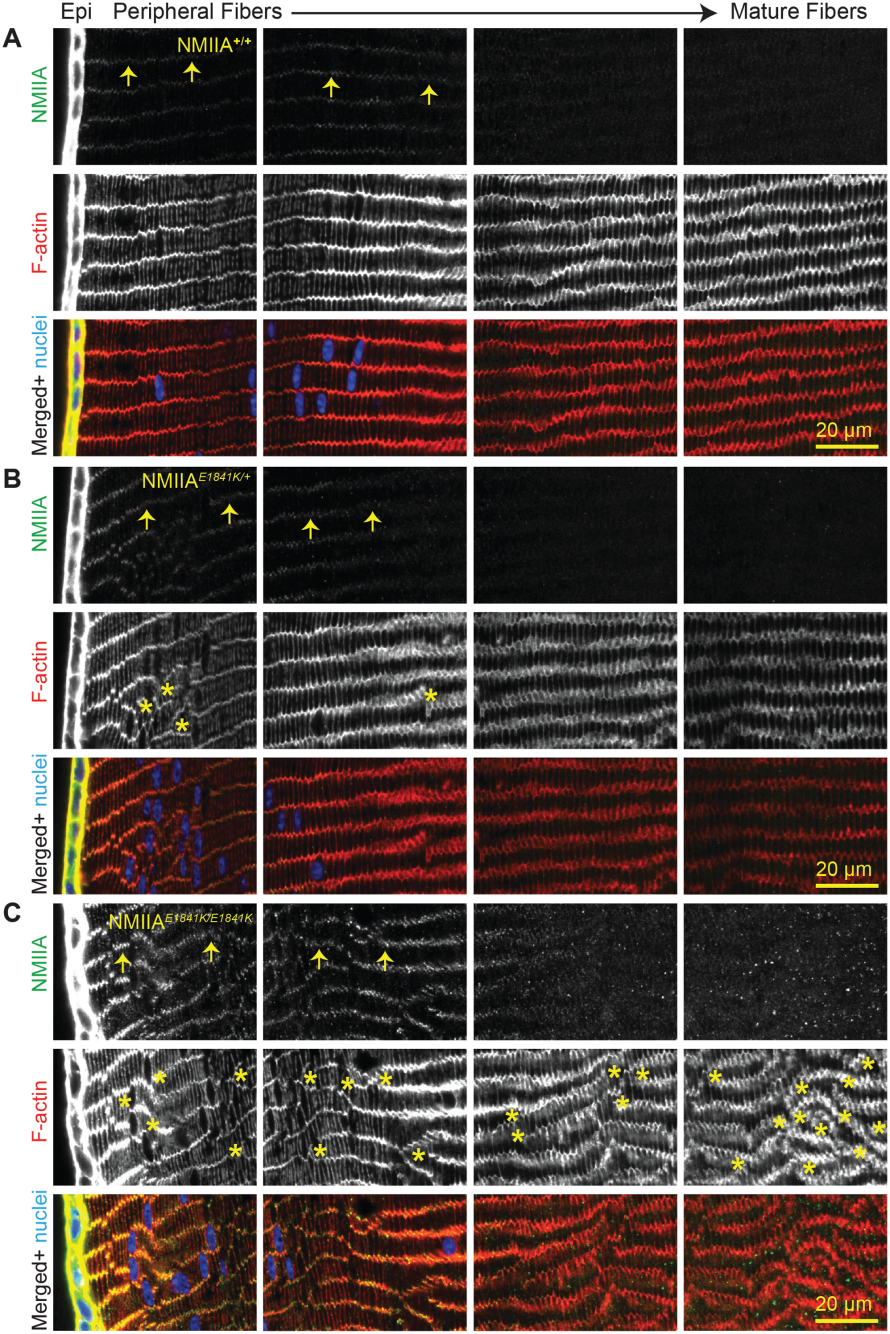
NMIIA is predominantly localized in lens epithelial cells of NMIIA*^+/+^*, NMIIA*^E1841K/+^*, and NMIIA*^E1841K/E1841K^* lenses. Immunostaining of frozen sections in the cross-orientation for (**A**) NMIIA*^+/+^*, (**B**) NMIIA*^E1841K/+^*, and (**C**) NMIIA*^E1841K/E1841K^* lenses for NMIIA (*green*), F-actin (*red*), and cell nuclei (*blue*). Images are equatorial sections, with sequential panels showing from left to right, the lens epithelium (Epi) and peripheral fiber cells (leftmost panel) inwards to the mature fiber cells (rightmost panel, approximately 260 µm deep). The first and second leftmost panels are 0 to approximately 65 µm deep and approximately 65 µm to approximately 130 µm deep, respectively. The mature fiber cells are seen in the third panel from the left (approximately 130 to approximately 195 µm deep) and rightmost panel (approximately 195 to approximately 260 µm deep). Although NMIIA is mostly localized to the lens epithelium, faint NMIIA puncta are present along the short vertices of the fiber cells (*arrows*). F-actin staining shows misaligned fiber cells in NMIIA*^E1841K/+^* and NMIIA*^E1841K/E1841K^* lens sections. Asterisks indicate regions of disorder. Scale bar, 20 µm.

To evaluate whether the NMIIA–E1841K mutation affects NMIIA localization, we immunolabeled equatorial cryosections from control and mutant lenses for NMIIA, F-actin, and nuclei. As reported previously,^39^^,^^40^ NMIIA is predominantly expressed in lens epithelial cells, with lower levels observed in fiber cells (Fig. 4; leftmost panel 0 to approximately 65 µm deep). In fiber cells, the NMIIA colocalizes with F-actin at the short vertices of the peripheral cortical fiber cells (Fig. 4, arrows). Similar NMIIA staining patterns are observed in NMIIA*^+/+^*, NMIIA*^E1841K/+^*, and NMIIA*^E1841K/E1841K^* lens sections, although NMIIA staining seems to be somewhat brighter in fiber cells of NMIIA*^E1841K/E1841K^* sections compared with fiber cells in NMIIA*^+/+^* and NMIIA*^E1841K/+^* sections (first and second panels from the left in Fig. 4, 0 to approximately 130 µm deep) (Figs. 4A, B, and C; arrows).

We also observed small NMIIA puncta in the cytoplasm of mature fiber cells of NMIIA*^E1841K/E1841K^* sections, which are not observed in NMIIA*^+/+^* and NMIIA*^E1841K/+^* lens sections (third panel from left, approximately 130 to approximately 195 µm deep; rightmost panel, approximately 195 to approximately 260 µm deep). In addition to the altered NMIIA staining patterns, we also noticed that NMIIA*^E1841K/+^* and NMIIA*^E1841K/E1841K^* lens sections have areas of disorder in the normally well-aligned radial columns of hexagonal lens fiber cells, which is most obvious in the homozygous mutant lens section (Figs. 4B and C, asterisks).

To visualize the weak NMIIA staining in fiber cells, it was necessary to saturate the NMIIA staining intensity in the epithelium. Therefore, we obtained lower intensity images of NMIIA staining in control and mutant lens sections (Supplementary Fig. S2). Similar to the fiber cells, the intensity of NMIIA staining is considerably brighter in the NMIIA*^E1841K/E1841K^* epithelial cells as compared with control and heterozygous epithelial cells. However, as shown in Figure 3, Western blots show no significant changes in the total NMIIA protein levels (Figs. 3A and B). This finding suggests that the NMIIA rod/tail domain epitope recognized by the antibody used for immunostaining may be more accessible to labeling in the NMIIA–E1841K molecules. It is possible that increased accessibility may be due to the abnormal bipolar filaments formed by the NMIIA–E1841K molecules.^55^

### Lens Fiber Cells Are Disorganized in NMIIA–E1841K Lenses

**Figure 5.**
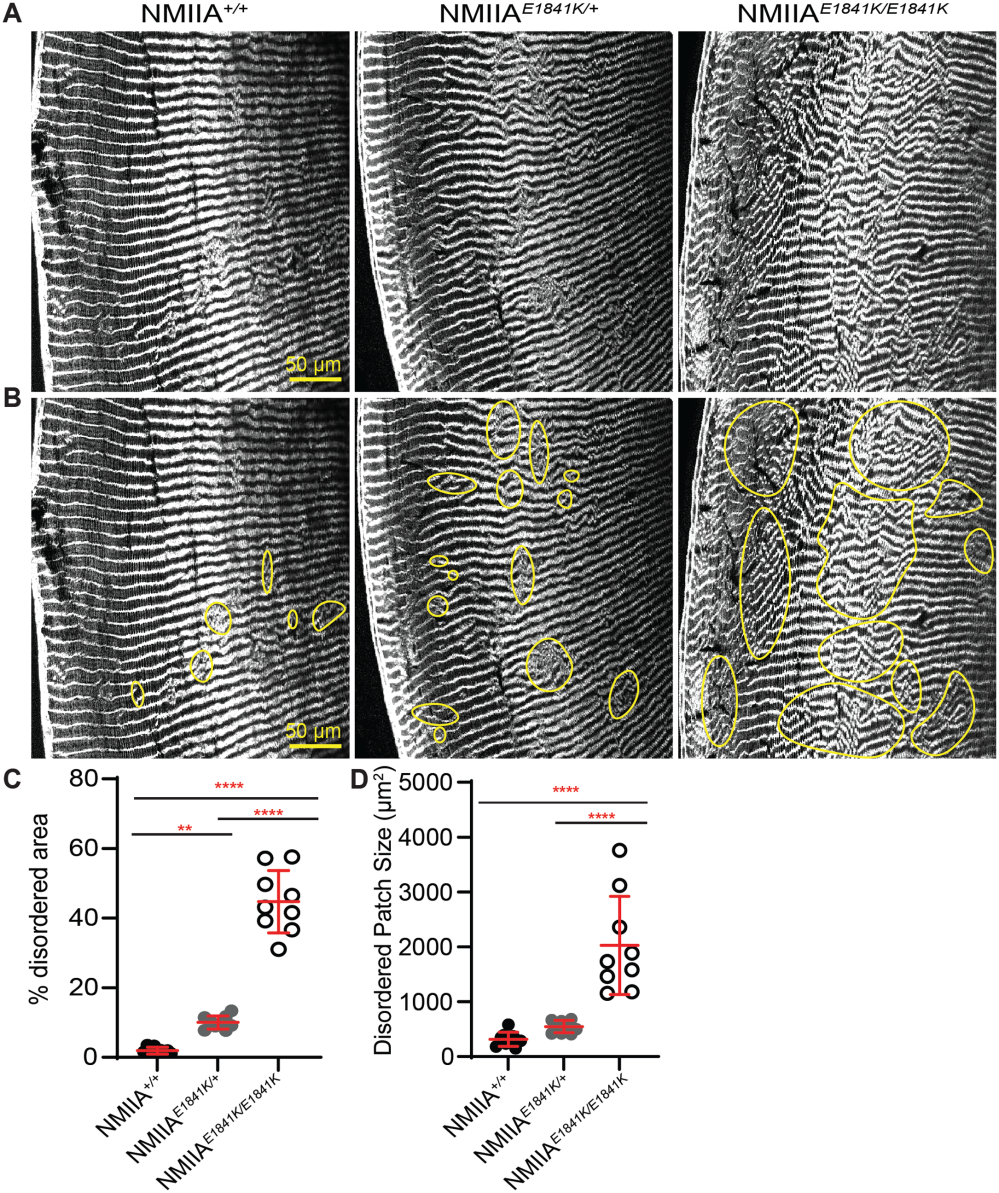
NMIIA*^E1841K/E1841K^* lenses display large areas of fiber cell disorganization. (**A**, **B**) Equatorial cryosections of NMIIA*^+/+^*, NMIIA*^E1841K/+^*, and NMIIA*^E1841K/E1841K^* lenses were immunolabeled with rhodamine-phalloidin (F-actin). (**A**) Cryosections without outlined disordered regions, whereas (**B**) shows regions of disorder outlined in yellow. Both NMIIA*^E1841K/+^* and NMIIA*^E1841K/E1841K^* lenses displayed more areas of fiber cell disorder as compared with *NMIIA^+/+^* lenses. Scale bar, 50 µm. (**C**) The percent disordered area was significantly greater in the NMIIA*^E1841K/+^* and NMIIA*^E1841K/E1841K^* lenses compared with NMIIA*^+/+^* lenses. (**D**) Disordered patch sizes were significantly higher in NMIIA*^E1841K/E1841K^* lenses compared with NMIIA*^+/+^* and NMIIA*^E1841K/+^* lenses, whereas the patch size is not significantly different between NMIIA*^+/+^* and NMIIA*^E1841K/+^* lenses. Plots reflect the mean ± SD of *n* = 9 independent immunostained sections from three different mice per genotype. **P < 0.01; ***P < 0.001; ****P < 0.0001.

We noticed that the lens fiber cells seemed to be disordered and displayed various degrees of misalignment in equatorial sections of NMIIA*^E1841K/E1841K^* lenses (Fig. 4C). To further evaluate the extent of disorder in the control versus mutant lenses, we obtained low-magnification images of large regions of equatorial cryosections stained for F-actin (Figs. 5A and B). Because F-actin is enriched at the fiber cell membranes,^61^^,^^65^^,^^70^ F-actin can be used as a marker for fiber cell boundaries, allowing an assessment of the cellular organization. We quantified the extent of disorder by outlining regions of disorder (Fig. 5B) and measuring their area relative to the section area, as previously described.^7^^,^^61^ The percent disordered area was very low at 1.9 ± 1.0% in NMIIA*^+/+^* lens sections (Fig. 5C). The disordered area percentage is significantly higher in both NMIIA*^E1841K/+^* lens sections (10 ± 1.9%) and in NMIIA*^E1841K/E1841K^* lens sections (44.7 ± 9.0%), as compared with controls (Fig. 5C). The average size of the disordered patches is also significantly greater in the NMIIA*^E1841K/E1841K^* lens sections (2026 ± 894.2 µm^2^) compared with either the NMIIA*^+^^/+^* lens sections (316.5 ± 128.6 µm^2^) or NMIIA*^E1841K/+^* lens sections (547.4 ± 111.7 µm^2^) (Fig. 5C). Although the average size of the disordered patches is somewhat greater in the lens sections from NMIIA*^E1841K/+^* heterozygous mice as compared with NMIIA*^+^^/+^* controls, this difference did not reach statistical significance.

**Figure 6.**
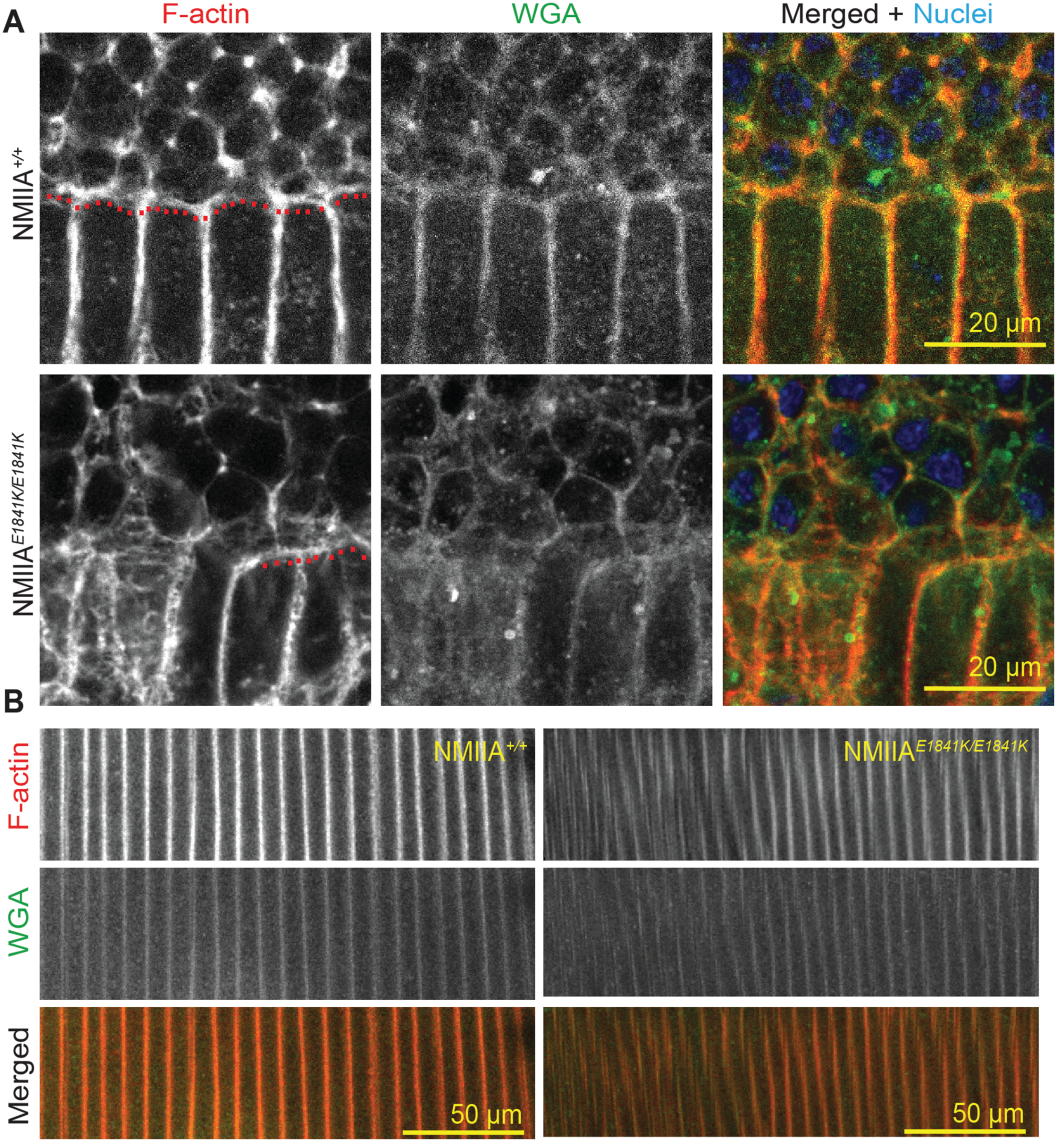
Peripheral fiber cells are disordered in the NMIIA*^E1841K/E1841K^* lenses. Whole fixed lenses were labeled with rhodamine-phalloidin (F-actin) (*red*), WGA (cell membrane, *green*), and nuclei (*blue*). (**A**) A single optical section of the peripheral fiber cells in the region of the lens fulcrum is shown in the XY plane. The fulcrum can be identified by a change in cell morphology and bright phalloidin staining (*red dashed line*). The fulcrum is irregular and discontinuous (short red dashed line) in the homozygous mutant lens, and peripheral fiber cells are not aligned in parallel rows in NMIIA*^E1841K/E1841K^* lenses in contrast to the NMIIA*^+/+^* lenses. Scale bars, 20 µm. (**B**) Images of a single optical section of peripheral fiber cells approximately 5.0 to 5.5 µm inwards from the fulcrum. The F-actin and WGA staining reveal irregularly spaced and misaligned fiber cells in the NMIIA*^E1841K/E1841K^* lens, while precisely aligned and regularly spaced fiber cell membranes are observed in the NMIIA*^+/+^* lens. Scale bars, 50 µm.

We also examined the organization of cortical fiber cells near the equator to determine whether newly formed fiber cells were disordered. We stained whole lenses from 6- to 8-week-old NMIIA*^+/+^* and NMIIA*^E1841K/E1841K^* mice for F-actin, wheat germ agglutinin (WGA) as a membrane marker,^71^ and nuclei, followed by whole mount z-stack confocal imaging at the lens equator. We observed that F-actin colocalizes with WGA at the cell membranes in both NMIIA*^+/+^* and NMIIA*^E1841K/E1841K^* lenses (Fig. 6). For depth standardization, we identified the fulcrum region of the lens, where the apical tips of elongating epithelial cells constrict to form an anchor point before fiber cell differentiation and elongation at the equator.^9^^,^^62^^,^^66^^,^^67^ The fulcrum is well-defined in NMIIA*^+/+^* lenses and appears as a continuous and relatively straight F-actin–enriched line at the equator (Fig. 6A, red dashed line). Although differentiating epithelial cells in NMIIA*^E1841K/E1841K^* lenses form a fulcrum that can still be located for depth standardization, the fulcrum is irregular and discontinuous (Fig. 6A, short red dashed line). Moreover, immediately below the fulcrum, the F-actin– and WGA–stained fiber cell membranes in the NMIIA*^E1841K/E1841K^* lens are not aligned vertically in regular parallel rows. The disrupted F-actin and WGA staining patterns suggest changes in the cell membranes and/or cell morphology in the NMIIA*^E1841K/E1841K^* lenses (Fig. 6A). We further examined the peripheral fiber cells located approximately 5 µm inward from the fulcrum (Fig. 6B). We observed vertically aligned, evenly spaced F-actin and WGA staining at fiber cell membranes in NMIIA*^+/+^* lenses, whereas misaligned and irregularly spaced F-actin and WGA staining were observed in the NMIIA*^E1841K/E1841K^* lenses, suggesting that the newly differentiating cortical fiber cells are disordered in the NMIIA–E1841K homozygous mutant lenses.

### Equatorial Epithelial Cells in Meridional Rows Are Misaligned in NMIIA^E1841K/E1841K^ Lenses

**Figure 7.**
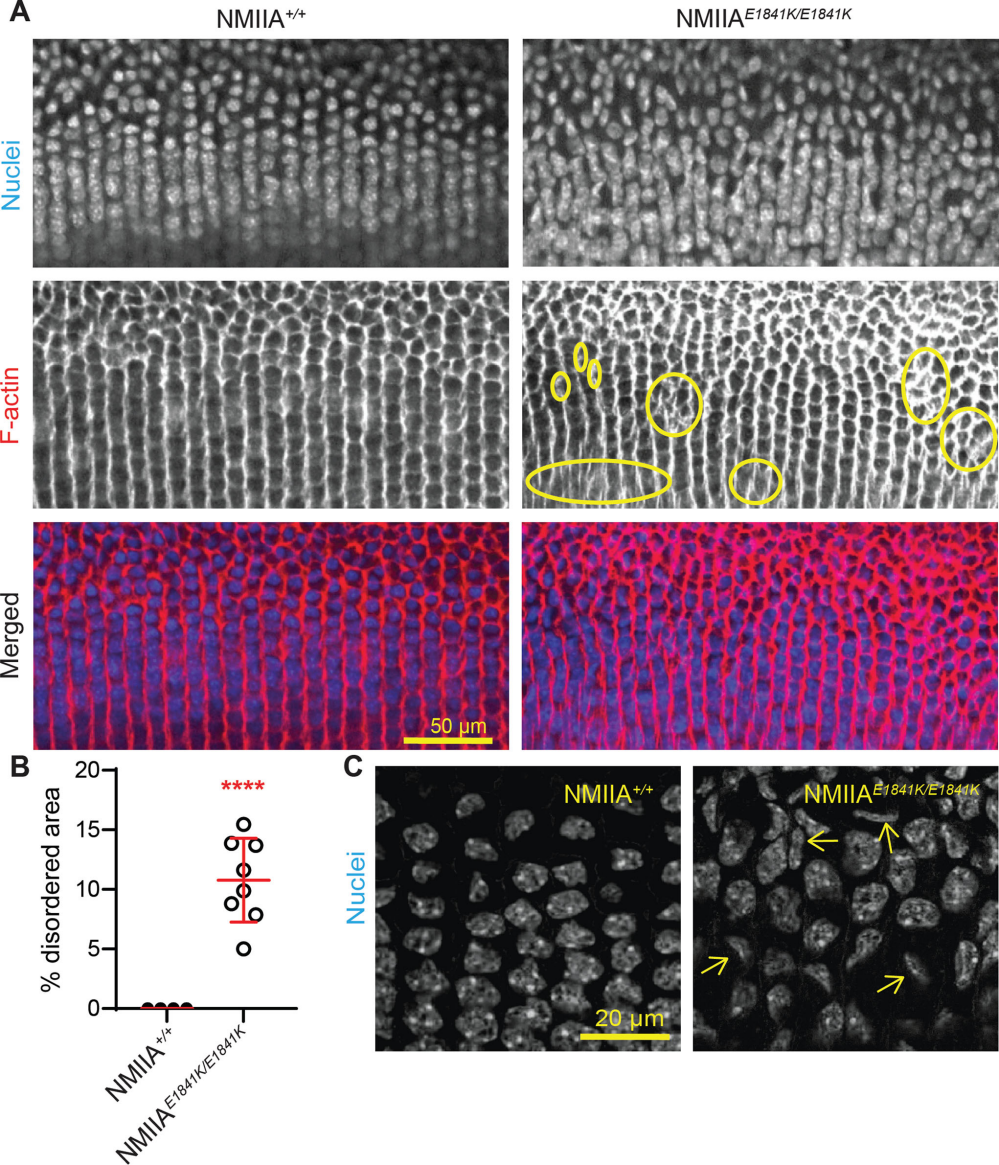
Equatorial epithelial cells in meridional rows are misaligned in NMIIA*^E1841K/E1841K^* lenses. (**A**) Whole mounts of fixed lenses from NMIIA*^+/+^* and NMIIA*^E1841K/E1841K^* mice labeled for nuclei (*top*) and F-actin (*middle*). Merged images (*bottom*) with nuclei in blue and F-actin in red. A single optical section in the XY plane displays the middle region of the meridional row cells at low magnification. Meridional row cells are hexagonal and precisely aligned, with nuclei arranged in parallel rows in the NMIIA*^+/+^* lens, whereas meridional rows are misaligned and branching (yellow circles), with disordered and misaligned nuclei in the NMIIA*^E1841K/E1841K^* lens. Scale bar, 50 µm. (**B**) Percent disordered area, plotted as the mean ± SD of four NMIIA*^+/+^* and eight NMIIA*^E1841K/E1841K^* lenses from at least four to five different mice. Each dot represents an individual lens, and there is a statistically significant increase in disordered area in homozygous mutant lenses compared with the control lenses. ****P < 0.0001. (**C**) High magnification view of meridional row cell nuclei in NMIIA*^+/+^* and NMIIA*^E1841K/E1841K^* lenses. A single optical section in the XY plane displays the nuclei at the mid-region of meridional row cells (i.e., middle of the lateral membrane with respect to apical–basal cell domains). The mid-region of the meridional row cells was identified based on the maximum diameter of the nuclei. The nuclei of the NMIIA*^E1841K/E1841K^* lens seem to be misaligned, out of plane, and abnormally shaped, in contrast with the aligned and regular pattern of nuclei in the NMIIA*^+/+^* lens. Scale bar, 20 µm.

Previous work showed that ordered packing of the lens fiber cells depends on the organization of hexagon-shaped equatorial epithelial cells into aligned meridional rows.^8^^,^^9^ We examined equatorial epithelial cells in the meridional row region for cell shape and alignment by whole-mount imaging of lenses stained for F-actin and nuclei. Imaging of the equatorial epithelial cells in control NMIIA*^+/+^* lenses at low magnification shows that initially disordered and randomly packed equatorial epithelial cells transform into precisely aligned meridional row cells as they move toward the lens equator (Fig. 7A). The meridional row epithelial cells in control lenses are hexagon shaped and well-aligned. In contrast, in NMIIA*^E1841K/E1841K^* lenses, we observe focal regions of meridional cell disorder with cellular misalignment and abnormal branching of rows (Fig. 7A, circles). The extent of the disorder in meridional row regions of NMIIA*^+/+^* and NMIIA*^E1841K/E1841K^* lenses was measured, revealing that the meridional rows of NMIIA*^E1841K/E1841K^* lenses exhibit an approximately 12% disordered area, with no measurable disorder in NMIIA*^+/+^* lenses (Fig. 7B). Nuclei are also precisely aligned and stacked above one another in the NMIIA*^+/+^* lens. However, in the NMIIA*^E1841K/E1841K^* lens, nuclei are misaligned and irregularly packed (Figs. 7A and C). Moreover, some of the nuclei appear deformed and distorted in NMIIA*^E1841K/E1841K^* lenses compared with control lenses (Fig. 7C, arrows).

### Loss of Regular Hexagonal Packing Is Observed in the Meridional Rows of NMIIA^E1841K/E1841K^ Lenses

**Figure 8.**
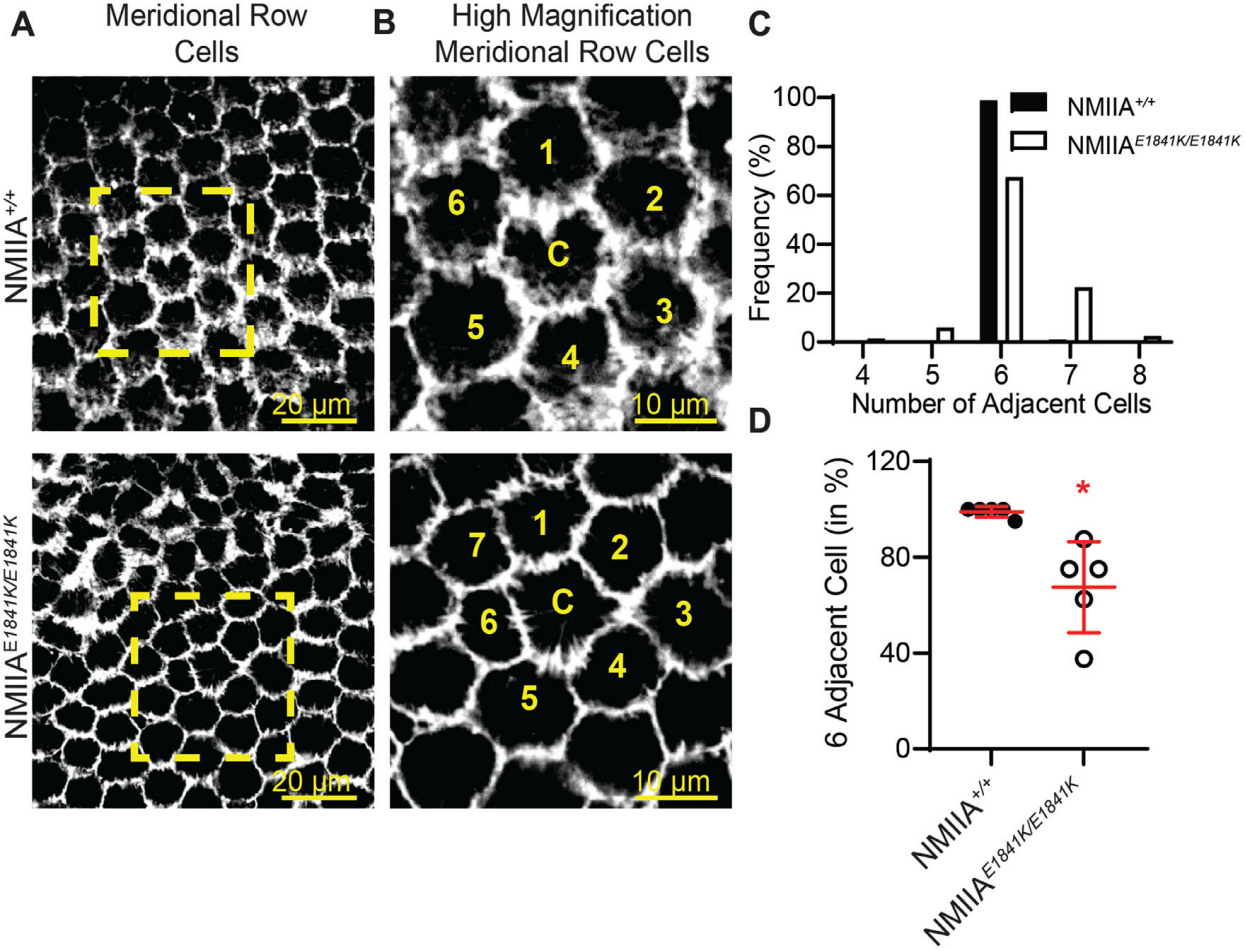
Cells in meridional rows exhibit aberrant cell shapes and irregular packing in NMIIA*^E1841K/E1841K^* lenses. (**A**) F-actin staining in single optical sections at the basal region of meridional row cells in NMIIA*^+/+^* and NMIIA*^E1841K/E1841K^* lenses, shown in XY plane. F-actin is enriched along all six sides of meridional row epithelial cells. Scale bar, 20 µm. *Yellow boxes*, regions enlarged in (**B**). (**B**) High magnification of a region from A (*yellow boxes*), showing an individual cell (**C**) (central cell) surrounded by six neighboring cells (numbered) in the NMIIA*^+/+^* lens, but surrounded by seven neighboring cells in the NMIIA*^E1841K/E1841K^* lens. Scale bar, 10 µm. (**C**) Frequency distribution (%) of the number of adjacent cells for 200 cells from 5 different lenses. Almost all cells in NMIIA*^+/+^* lenses have six nearest neighbors, whereas cells in NMIIA*^E1841K/E1841K^* lenses have variable numbers of nearest neighbors (4–8). (**D**) Percentage of hexagonal adjacent cells in NMIIA*^+/+^* and NMIIA*^E1841K/E1841K^* lenses. The plot represents the mean ± SD of five different lens images from at least four different mice. *P < 0.05.

We next used whole-mount imaging of lenses to examine the meridional row cells at high magnification to investigate the extent of hexagonal cell packing in NMIIA*^E1841K/E1841K^* lenses. F-actin staining was used to assess cell shapes and packing, since it is enriched around the entire perimeter of meridional row cells and at all six vertices of the basal regions of cell membranes in the NMIIA*^+/+^* lens (Figs. 8A and B). Although the organized meridional row cells in NMIIA*^+/+^* lenses are hexagon shaped and similar in size, the shapes of some of the meridional row cells in the NMIIA*^E1841K/E1841K^* mutant lens are distorted and not hexagon shaped (Figs. 8A and B). Compared with control cells, mutant cells are asymmetrical with varying cell shapes and sizes, leading to defective packing organization. In control lenses, a single hexagon-shaped meridional row cell is normally surrounded by six other cells (Figs. 8B–D). The average percentage of cells with six neighbors and the frequency distribution analysis show 99% of NMIIA*^+/+^* cells have six neighboring cells, whereas only 67.5% of the NMIIA*^E1841K/E1841K^* cells have six neighboring cells (Figs. 8C and D). In addition, the number of neighbors for NMIIA*^E1841K/E1841K^* cells is highly variable compared with a consistent six neighbors for NMIIA*^+/+^* cells (coefficient of variation: NMIIA*^+/+^* = 2.3% and NMIIA*^E1841K/E1841K^* = 28%).

## Discussion

Here, we have identified a novel function of NMIIA in regulating the alignment and hexagonal packing of lens meridional row epithelial cells and fiber cells. The NMIIA–E1841K mutation disrupts the formation of neat meridional rows by equatorial epithelial cells and the transition of meridional row cells to form organized fiber cells (Figs. 4^5^,^6^,^7^–8). Consistent with previous studies,^8^^,^^9^ the alignment of meridional row epithelial cells is required for subsequent fiber cell organization. Our work demonstrates that proper NMIIA bipolar filament assembly regulates the precise alignment of lens epithelial cells into meridional rows during secondary fiber cell differentiation.

The establishment of hexagonal honeycomb packing in meridional row epithelial cells at the equator is the genesis of the hexagonal packing observed in secondary fiber cells (i.e., hexagonal profiles of secondary fiber cells in cross-section).^72^ Previous work indicates that a loss of hexagonal packing in secondary fiber cells can occur in two possible ways. First, the precursor meridional row epithelial cells are disordered during initial differentiation with disorder persisting in newly formed fiber cells and maintained throughout their continued differentiation and elongation, as observed in *EphA2^−^^/^^−^* and *Src^−^^/^^−^* lenses.^8^^,^^9^ Alternatively, despite normal organization of meridional row cells, cortical fiber cells can become disordered during subsequent elongation and maturation as seen in *Tmod1^−^^/^^−^;CP49^−^^/^^−^* lenses.^7^^,^^61^ Our analysis of the extent of disordered area in NMIIA*^E1841K/E1841K^* lenses reveals that approximately 12% of the meridional row cells are disorganized whereas approximately 44.7% of the fiber cells are disorganized. Because NMIIA is expressed predominantly in the lens epithelial cells^39^ (Fig. 4), it is likely that the disorder is initiated in meridional row cells, which persists as the secondary fiber cells differentiate and elongate in NMIIA-E1841K lenses. The increased extent of fiber cell disorder may be explained by the process of fiber cell formation in which new layers of disordered fiber cells are continuously added on top of previous layers of disordered cells, leading to a greater extent of disorder. Alternatively, or additionally, the expression of NMIIA-E1841K mutant proteins in fiber cells during elongation could result in further disordered packing by interfering with normal fiber cell–cell interactions. It is thought that F-actin:NMII:N-cadherin complexes at the posterior basal surface of the elongating fiber cells transmit equal contractile tension between adjacent cells to stabilize the hexagonal cell shape, which may be required for appropriate cell packing, migration, and alignment of differentiating fiber cells.^2^^,^^73^ The abnormal NMIIA–E1841K filaments could disrupt the normal organization of F-actin:NMII:N-cadherin complexes and the balance of forces between cells, leading to irregular and disordered fiber cell packing.

The NMIIA–E1841K mutation in the coiled–coil rod domain alters lateral associations of the NMIIA rod domain^74^^,^^75^ such that myosin heads (motors) project all along the length of bipolar filaments leading to the loss of the central bare zone.^55^ NMIIA–E1841K mutant bipolar filaments are also significantly thicker and wider.^55^ These structural changes in NMIIA filaments caused by the E1841K mutation result in aberrant cellular actomyosin organization in mouse megakaryocytes, Sertoli cells, and primary podocytes.^50^^,^^55^^,^^76^ We speculate that abnormal NMIIA–E1841K filaments also result in altered actomyosin networks in *NMIIA^E1841K/E1841K^* lens epithelial cells, resulting in distorted cell shapes, misalignment, and irregular packing arrangements of the meridional row cells in *NMIIA^E1841K/E1841K^* lenses. Because Western blots show that the E1841K mutation does not affect total NMIIA protein levels, the brighter NMIIA staining observed in the *NMIIA^E1841K/E1841K^* lenses (Fig. 4) is most likely a consequence of increased antibody access to an antigenic site, consistent with structural changes in NMIIA bipolar filaments owing to the E1841K mutation.^55^ NMIIA puncta are also only seen in *NMIIA^E1841K/E1841K^* lens sections and could indicate NMIIA aggregation, consistent with abnormal NMIIA filament assembly. These structural changes in NMIIA could also contribute to the loss of actomyosin function during lens epithelial cell alignment and hexagonal packing at the equator.

The only two proteins that are known to regulate meridional row alignment are EphA2 and Src.^8^^,^^9^ EphA2 binds and phosphorylates Src,^13^ which then activates cortactin to recruit the actin cytoskeleton to cell–cell junctions and enables the lens epithelial cells to form aligned meridional rows.^9^^,^^13^ Previous studies show that *EphA2^−^^/^^−^* and *Src^−^^/^^−^* lenses exhibited disruption of meridional row cell shape and packing, along with subsequent fiber cell alignment, similar to *NMIIA^E1841K/E1841K^* lenses.^8^ EphA2 signaling pathway regulates Src and RhoA-GTPase, which then activates Rho-associated protein kinase to phosphorylate myosin light chain (increasing NMII activity).^77^^–^^79^ Therefore, NMIIA is a potential downstream target of the EphA2 and Src signaling cascade, which could explain the similarity in phenotype between *EphA2^–/–^*, *Src^–/–^*, and *NMIIA^E1841K/E1841K^* lenses. However, in *NMIIA^E1841K/E1841K^* lenses, the shapes of cell nuclei seem to be more distorted, tilted, and misshapen than in the EphA2 or Src knockout lenses. Other actin-associated proteins and cell–cell adhesion proteins such as periaxin, Abi2, E/N-cadherin, β-catenins, and Arvcf (Armadillo repeat gene deleted in velocardiofacial syndrome) have also been implicated in secondary fiber cell organization,^80^^–^^85^ but the etiology of meridional row and secondary fiber cell disorder in these knockout mice has either not been studied or cannot be investigated due to the severity of the lens defects. Defects in secondary fiber cell elongation and fulcrum formation have been observed in aPKCλ (cell polarity protein) knockout mouse lenses, although meridional row cells were not examined in detail.^86^ Further investigation is required to understand the interplay between Eph–ephrin signaling, other actin-associated proteins, and NMIIA in regulating equatorial lens epithelial and fiber cell alignment and packing.

Our studies overall indicate that fiber cell misalignment and irregular hexagonal packing in the NMIIA–E1841K lenses do not contribute to whole lens morphology, transparency, or stiffness. Fiber cell misalignment and irregular packing do not affect lens transparency in the 2-month-old *Tmod1^−^^/^^−^;CP49^−^^/^^−^* mice.^61^ In aging mouse lenses, the fiber cells near the lens periphery are disordered, yet the lens outer cortex of old lenses is transparent.^37^ This finding indicates that precise hexagonal packing is not required for lens transparency. In addition, irregular lens meridional row and fiber cell organization in *EphA2^–^^/^^–^* mice do not affect whole lens stiffness,^87^ as also observed in NMIIA–E1841K mutant mouse lenses (Supplementary Fig. S1). Previous work has shown that pharmacological inhibition of myosin II contractile activity decreases whole lens stiffness in 7-day-old chickens.^68^ The difference from our findings could be due to functional compensation by NMIIB^88^ being sufficient to maintain lens stiffness in NMIIA–E1841K mutant lenses, whereas the pharmacological inhibition of all myosin isoforms would prevent compensation by other isoforms of NMII.

Between 16% and 18% of *MYH9*-RD patients present with presenile cataracts with a mean onset age of 23 to 37 years old.^41^^,^^89^ Genotype–phenotype correlation studies have categorized the patients with the E1841K substitution (*n* = 31 patients) as having a low risk of cataract development^41^; however, owing to the low number of patients evaluated, the actual incidence of cataracts associated with these mutations may not be represented in this study. We observed that some NMIIA*^E1841K/+^* mice in the mixed FvBN/129SvEv/C57BL6 at 6 months^53^ presented with opacities before the backcrossing of mutant mice with C57BL/6J wild-type mice. Cataract phenotype severity and variations in incidence due to mouse strain variability have been observed previously in connexin 50,^90^ connexin 46,^91^ and ephrin-A5^8^^,^^12^^,^^92^^–^^94^ knockout mice. Strain variability could explain why a cataract phenotype in *Myh9-*RD mutant mice disappeared upon backcrossing. We would also expect *MYH9-*RD human patients from different populations to have different cataract phenotypes and incidences as well. In future studies, it will be interesting to evaluate whether mice with the NMIIA–E1841K mutation may develop early onset cataracts with aging.

In conclusion, NMIIA regulates meridional row alignment and hexagonal cell packing, which in turn contributes to normal fiber cell morphogenesis during lens differentiation and establishes subsequent hexagonal packing of mature fiber cells. The location of the NMIIA–E1841K mutation in the NMIIA rod domain indicates that normal bipolar filament assembly of NMIIA is required for meridional row alignment and hexagonal packing. To further elucidate how actomyosin remodeling promotes cell shape transformation and precise alignment in the lens, it will be important to investigate NMIIA:F-actin network organization before, during, and after cell shape changes during formation of meridional rows at the lens equator.

## Supplementary Material

Supplement 1
