# Supplementary material for: Nonmuscle Myosin IIA Regulates the Precise Alignment of Hexagonal Eye Lens Epithelial Cells During Fiber Cell Formation and Differentiation
Source: Invest Ophthalmol Vis Sci. 2023 Apr 18;64(4):20. doi: 10.1167/iovs.64.4.20 (PMC10123325; doi:10.1167/iovs.64.4.20)
Supplement: Supplement 1 [file iovs-64-4-20_s001.pdf]

NMIIA$^{E1841K/+}$ X NMIIA$^{E1841K/+}$			
Genotype	Total	Male	Female
NMIIA$^{+/+}$	145	76	69
NMIIA$^{E1841K/+}$	219	117	102
NMIIA$^{E1841K/E1841K}$	49	25	24

Supplementary Table 1. Genotypes of offspring from heterozygous mutant mouse breedings. Male and female NMIIA$^{E1841K/+}$ mice were interbred to generate NMIIA$^{+/+}$, NMIIA$^{E1841K/+,}$ and NMIIA$^{E1841K/E1841k}$ mice. Out of 413 pups in the E1841K strain, 35% were NMIIA$^{+/+}$, 53% were heterozygous and 12% were homozygous mutant mice.

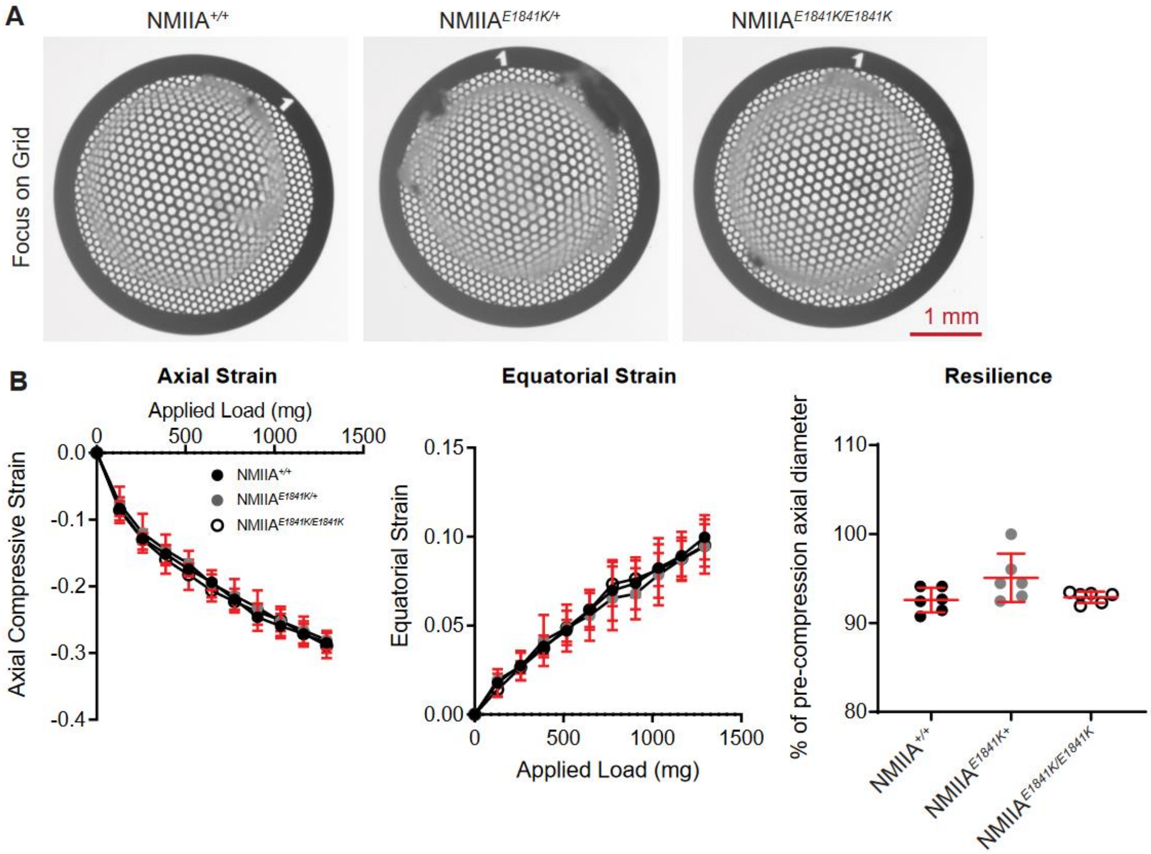

Supplementary Figure 1. NMIIA-E1841K mutation does not affect lens focusing or biomechanics. (A) Top-view images of freshly dissected 2 months old NMIIA$^{+/+}$, NMIIA$^{E1841K/+}$, and NMIIA$^{E1841K/E1841K}$ lenses on electron microscopy grids. There is no apparent difference in lens focusing between wildtype and NMIIA-E1841K mutant lenses. Scale bars, 1 mm. (B) Compression testing of 2 months old control and NMIIA-E1841K mutant lenses show no significant changes in axial compressive strain or equatorial expansion strain. Lens resilience, calculated as the ratio of the pre-compression over post-compression axial diameter, also is unaffected by either mutation. Plots reflect mean ± SD of 6 lenses from 3 biological replicates per genotype.

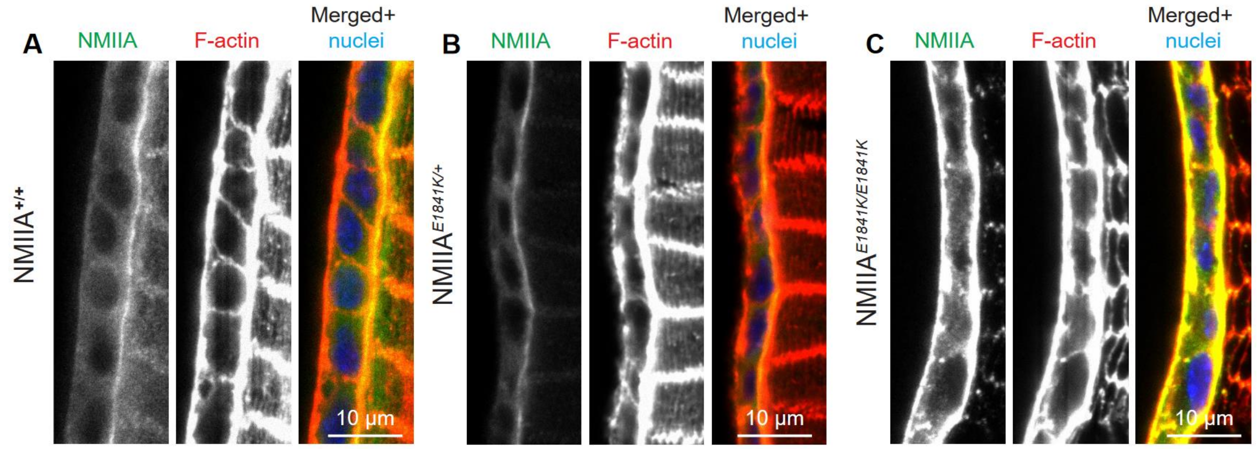

Supplementary Figure 2. NMIIA is enriched at the lens epithelial cells of NMIIA$^{+/+}$, NMIIA$^{E1841K/+}$, and NMIIA$^{E1841K/E1841K}$ lenses. Brighter NMIIA staining is observed in the lens epithelial cells of NMIIA$^{E1841K/E1841K}$ lenses compared to NMIIA$^{+/+}$ and NMIIA$^{E1841K/+}$ lens sections (A, B, C) Immunostaining of frozen sections in the cross-orientation of (A) NMIIA$^{+/+}$, (B) NMIIA$^{E1841K/+}$, and (C) NMIIA$^{E1841K/E1841K}$ lenses for NMIIA (green), F-actin (red), and cell nuclei (blue). Zoomed-in images of equatorial lens epithelium with low intensity NMIIA staining (Note that Fig. 4 shows saturated NMIIA staining in the epithelium to visualize the weak NMIIA staining in fiber cells). . Scale bar, 10μm.
